# Neuroimaging and natural language processing-based classification of suicidal thoughts in major depressive disorder

**DOI:** 10.1038/s41398-024-02989-7

**Published:** 2024-07-04

**Authors:** Dong Yun Lee, Gihwan Byeon, Narae Kim, Sang Joon Son, Rae Woong Park, Bumhee Park

**Affiliations:** 1 Department of Biomedical Informatics, Ajou University School of Medicine, Suwon, South Korea; 2 Department of Medical Sciences, Graduate School of Ajou University, Suwon, Republic of Korea; 3 Department of Psychiatry, Kangwon National University School of Medicine, Chuncheon, Republic of Korea; 4 Department of Biomedical Sciences, Ajou University Graduate School of Medicine, Suwon, Republic of Korea; 5 Department of Psychiatry, Ajou University School of Medicine, Suwon, South Korea; 6 Office of Biostatistics, Medical Research Collaborating Center, Ajou Research Institute for innovative medicine, Ajou University Medical Center, Suwon, Republic of Korea

**Keywords:** Diagnostic markers, Depression

## Abstract

Suicide is a growing public health problem around the world. The most important risk factor for suicide is underlying psychiatric illness, especially depression. Detailed classification of suicide in patients with depression can greatly enhance personalized suicide control efforts. This study used unstructured psychiatric charts and brain magnetic resonance imaging (MRI) records from a psychiatric outpatient clinic to develop a machine learning-based suicidal thought classification model. The study included 152 patients with new depressive episodes for development and 58 patients from a geographically different hospital for validation. We developed an eXtreme Gradient Boosting (XGBoost)-based classification models according to the combined types of data: independent components-map weightings from brain T1-weighted MRI and topic probabilities from clinical notes. Specifically, we used 5 psychiatric symptom topics and 5 brain networks for models. Anxiety and somatic symptoms topics were significantly more common in the suicidal group, and there were group differences in the default mode and cortical midline networks. The clinical symptoms plus structural brain patterns model had the highest area under the receiver operating characteristic curve (0.794) versus the clinical notes only and brain MRI only models (0.748 and 0.738, respectively). The results were consistent across performance metrics and external validation. Our findings suggest that focusing on personalized neuroimaging and natural language processing variables improves evaluation of suicidal thoughts.

## Introduction

According to the World Health Organization, the suicide mortality rate is approximately 10.7 per 100,000 people. It is the second leading cause of death in young people [1]. And the prevalence of suicide is gradually increasing. From 1999 through 2018, the suicide rate increased 35%, from 10.5 to 14.2 per 100,000 in United States [2]. Similarly in South Korea, the disease burden due to suicide has gradually increased, reaching 1.7 times the burden (years of life lost) measured from 2000 to 2018 [3]. Consequently, suicide has gradually become more important as a social problem, so it is very important to predict the risk of suicide in advance to prevent it.

The most important risk factor for suicide suggested in previous epidemiological studies is an underlying psychiatric illness, especially depression, substance use disorder, and psychosis [4]. According to a recently published narrative review study, the association with suicidal behavior was more significant for specific symptom dimensions than for a diagnosis of mental illness [5]. To support this, anhedonia, psychological pain, and psychotic experiences have been shown to predict suicidal behavior independently of the underlying psychiatric disorder [6–8]. These research results show that both the diagnosis and detailed symptoms must be considered when assessing the suicide risk of patients in clinical settings. A good data source for checking specific symptoms is the medical records that psychiatrists write while providing treatment. However, because these records are qualitative, it has been difficult to conduct quantitative analysis. A method that can overcome these limitations is automated natural language processing (NLP), which was recently introduced. Using NLP, words can be derived from qualitative records to generate quantitative data [9], and features related to the patient’s prognosis can be calculated from qualitative records, such as medical records [10]. Similarly, it could be possible to statistically derive a patient’s detailed psychopathological symptoms from medical records and determine if they can classify suicidal thoughts.

Additionally, brain magnetic resonance imaging (MRI) is actively used in psychiatric clinical settings to differentiate organic causes. However, psychiatric diseases, such as mood disorders, often do not have visible brain lesions, so it is difficult to confirm significant changes through qualitative image analysis. To overcome this problem, methods are being attempted to predict the risk of suicide by measuring structural regional brain volume using quantitative image analysis techniques [11]. Recently, along with regional volume, relative structural volume patterns among brain network have been significantly associated with mental illness [12, 13]. Considering previous studies that the brain’s structural pattern could classify the presence or absence of suicidal thoughts [14], MRI data potentially can be used as one biomarker for suicide.

Concealment of suicidal ideas or attempts in psychiatric clinical settings is common. A study conducted in Australia reported that only 25% of patients who visited the clinic disclosed suicidal ideas [15]. If suicidal probability can be modeled through symptoms in medical records and brain MRI, then it would be able to screen suicidal thoughts early when they are not directly reported or hidden.

With this background, we aimed to classify suicidal thoughts at the time of depression diagnosis by leveraging brain morphometry and NLP. We also determined which psychiatric symptoms and structural brain volume patterns in the model were most important in classifying suicidal thought.

## Methods

This study was conducted in compliance with ethical standards depicted in the Helsinki declaration and approved by the Institutional Review Board of the Ajou University Hospital (AJOUIRB-DB-2022-335). Informed consent was not required owing to the use of de-identified data.

### Data sources

For the model development, patients who visited the Department of Psychiatry and Mental Health Center at the Ajou University School of Medicine (AUSOM) in South Korea between 2010 and 2022 were included in the study [16]. For the external validation, patients who visited the Department of Psychiatry and Mental Health Center at the Kangwon National University Hospital (KNUH) in South Korea between 2015 and 2022 were included in the study. The clinical data including socio-demographics, diagnoses, observations, provider visits, procedures performed, medications filled, clinical notes, and brain MRI were obtained. We identified patients with a new depressive episode for the study population. The index date was defined as the time of the patient’s first diagnosis of depressive disorder. To avoid any bias from left-censored data and to verify their first diagnosis of depressive disorder, we excluded patients who had been enrolled in the database for <1 year before the index date. Patients who had a diagnosis of bipolar disorder, schizophrenia, and/or dementia were also excluded. Patients who underwent brain MRI scans within 1 month of their first diagnosis were included. The final data of 210 patients were in accordance with the study criteria. Further details regarding code lists are described in Supplementary Table 1. The databases were formatted according to the Observational Medical Outcomes Partnership–Common Data Model version 5.3.1, maintained by the Observational Health Data Sciences and Informatics (OHDSI) and de-identified [17].

### Study design and outcome

To screen suicidal thoughts at an early stage, we developed models to classify the risk of suicidal thoughts in patients with the first diagnosis of depressive disorder using the extreme gradient boosting (XGBoost) algorithm. Suicidal thoughts at the index date were used as the primary outcome for the classification model. To identify suicidal thoughts, we used the initial record by psychiatrists recorded at the time of the depression diagnosis. The psychiatrist asks about suicidal ideation, suicide plans, and suicide attempts during the initial interview and records them on the initial interview sheet. We defined suicidal thoughts as the presence of any of the following in the initial record: suicidal ideation, planning, or attempts.

### Topic modeling

We used the psychiatrist’s initial notes at the time of depression diagnosis for the topic modeling. In the initial record, the main symptoms were written in the chief complaint section, and these symptoms included only those that are in the DSM-5, and all of them were written in English. We did a regular expression to extract chief complaints for NLP. Subsequently, NLP algorithms were used to extract topics as predictive variables from each patient’s chief complaints. Specifically, we converted the patient’s chief complaints into a bag-of-words model of the corpus after stemming, normalization, and stop-word removal. Latent Dirichlet allocation (LDA) as an unsupervised learning method was used to cluster the topics from each patient [18]. With an LDA-based topic model, the topic probabilities were calculated for each chief complaint. For instance, if five topics were created by the LDA from chief complaints, the probability of being assigned to five topics for each chief complaint was generated [19]. Prior to the use of LDA, we also calculated the perplexity scores in order to determine the optimal number of topics in the LDA [20]. The perplexity score can be an estimate of the relative quality of statistical models [21]. Specifically, Griffiths2004 and Deveaud2014 represent maximization approaches which means that a higher numerical fit score implies a better fit. CaoJuan2009 and Arun 2010 represent minimization approaches which means that a lower numerical fit score implies a better fit. We selected five as the number of topics based on the perplexity scores (Supplementary Fig. 1).

### Source-based morphometry analysis

For considering regional volume information in our model, structural T1-weighted MRI data from 1.5 T or 3 T scanners at AUSOM (Signa HDx 1.5 T and Discovery MR750w 3 T, respectively; GE Healthcare, Milwaukee, MI, USA) were collected from all participants. The T1-weighted image acquisitions used a spin-echo sequence with specific scan parameters for each scanner. Further details regarding scan parameters are described in the Supplementary Table 2. Additionally, for the external validation, structural T1-weighted MRI data from 1.5 T or 3 T scanners at KNUH (Magnetom Avanto 1.5 T, Siemens Healthcare, Erlangen, Germany; Achieva dStream 3.0 T, Philips Medical Systems, Best, Netherlands, respectively) were collected from all participants. The T1-weighted image acquisitions used a spin-echo sequence with specific scan parameters for each scanner (Supplementary Table 2). All MRI images were visually inspected by neuroradiologists, and the radiology reports were reviewed by psychiatrists. No observable scanning artifacts or gross brain abnormalities were identified in any participant included in the following analyses.

All of the structural MRI scans were used to estimate spatially independent morphologies as common patterns of gray matter concentration across all of the subjects using SBM approach [22]. Specifically, we applied cross-sectional independent component analysis (ICA) to data preprocessed using voxel-based morphometry (VBM), which is typically used to measure voxel-level volumetric maps [23]. VBM processing was performed using the SPM12 VBM-Diffeomorphic Anatomical Registration Through Exponentiated Lie Algebra (DARTEL) method [24]. This procedure involved segmenting gray matter from T1-weighted images based on a standard tissue probability map, creating a study-specific template, spatially normalizing individual images to the DARTEL template, modulating to adjust for volume signal variations, and spatially smoothing gray matter slices using a 6 mm full-width at half maximum Gaussian kernel. We applied a FastICA + ICASSO framework to the VBM preprocessed data after estimating individual VBM maps [25]. Laplace principal component analysis was used to reduce the original data to the optimal number of principal components. ICASSO was used to compute hierarchical clustering according to the dissimilarities between independent components (ICs) in each run after FastICA was run 100 times on the reduced data with random initial values [26–28]. Among the ICASSO results, meaningful IC maps with high reliability >0.8 were visually identified. All group-level IC maps were z-scored and thresholded with z > 3 for visualization. Finally, the IC maps that passed the threshold were selected as the patients’ brain networks.

### Model development and evaluation

Baseline characteristics were compared by presence or absence of suicidal thoughts within the database and across databases using independent samples t-tests and chi-square analyses for parametric and non-parametric variables, respectively. Pearson’s correlation between MRI variables and text variables was evaluated before developing models. Using the correlation matrix, we considered that indicators with r-values of >0.7 had multicollinearity [29]. *P*-values < 0.05 were considered statistically significant.

In this study, we developed classification models for suicide according to the combination of data types: weights of IC maps from brain MRI scans and the topic probabilities from clinical notes. Three models including MRI only, text only, and combining MRI and text were developed. The XGBoost, which can produce accurate predictions in the medical field, was used to develop all models [30]. The eligible patient cohort from AUSOM was split into a 75% training set and a 25% test set (internal validation) stratified by the proportion of patients with suicidal thoughts. During the model creation process, fivefold cross-validation on the training set was used for each classification model to tune hyperparameters, such as tree depth, learning rate, and number of trees [31]. A grid search using the AUROC was performed to optimize the hyperparameters of each model. The hyperparameters used and values are shown in Supplementary Table 3. Once the hyperparameters were selected and the best-performing cross-validated model was retrained on the entire training set, the best-performing model was evaluated on the 25% held-out test set. The AUROC, accuracy, sensitivity, specificity, and F1 score were calculated to evaluate the performance of the models using the test dataset. We used the maximal Youden index to select the optimal cutoff value in the prediction model [32].

### External validation

We used the KNUH database to perform external validation. The process of external validation was performed from the feature extraction stage. The topic probabilities obtained through LDA at AUSOM were extracted from patients at KNUH. Specifically, after preprocessing chief complaints of KNUH patients, the word probabilities of each topic from AUSOM were used to calculate the topic probabilities of each patient (Supplementary Fig. 2A). The brain networks obtained through SBM at AUSOM were also extracted from patients at KNUH. Specifically, after preprocessing the brain images of KNUH patients, the brain network data of AUSOM was projected onto the KNUH patient data to obtain the values (Supplementary Fig. 2B).

After extracting the features from the KNUH database, trained models from the AUSOM database were evaluated by the KNUH database. Evaluations were performed in the same manner used in the development stage.

### Comparison models

The model performance, using only MRI variables and using only text variables, was compared to that of both variables combined. After comparing the internal validation performance of the models, we compared the internal and external validation performance by model. DeLong’s tests were performed to statistically compare the AUROCs between models [33]. We also compared other metrics including accuracy, sensitivity, specificity, and F1 score.

### Model interpretation and visualization

Shapley Additive Explanations (SHAP) values were used to present the feature importance of the classification model (Supplementary Fig. 3). The effect of each feature on suicide was presented as a SHAP value representing the importance of a variable by deriving a marginal distribution and weighted average with all but the variable of interest fixed [34]. The SHAP summary plot sorts features in descending order on the basis of the effects on suicide. Each dot on each variable line represents one patient, and the horizontal location indicates the level of association between the feature and outcome. The right side shows the SHAP values > 0, and variable-specific SHAP values > 0 indicate an increased risk of outcome.

### Software

All analyses except brain MRI scans were performed using R software version 4.1.0 (R Foundation for Statistical Computing, Vienna, Austria), OHDSI’s Health Analytics Data to Evidence Suite packages, and open-source statistical R packages. For brain MRI scans, statistical analyses were performed using MATLAB (MathWorks, Sherborn, MA, USA) (“MATLAB - MathWorks - MATLAB &Simulink,” n.d.)-based custom software.

## Results

### Demographic and clinical characteristics

For model development, we included 152 patients with a new depressive episode from the AUSOM in South Korea. For model validation, we studied 58 patients with a new depressive episode from KNUH in South Korea. Table 1 and Supplementary Table 4 show the demographic and clinical characteristics of the study population. A total of 210 patients were included in the study, of which 126 (60%) were female and 84 (40%) were male. Among all included patients, the mean patient age was 55.2 years. Among the 152 patients in the AUSOM database, 36 (23.7%) reported suicidal thoughts when their depression was diagnosed. In the KNUH database, 18 (31.0%) among 58 patients reported suicidal thoughts when diagnosed with depression. When comparing the two institutions, the mean patient age at AUSOM was 55.9 years (SD, 17.6 years) and at KNUH was 53.7 years (SD, 23.4 years), with no significant difference (*p* = 0.523). However, patients who reported suicidal thoughts had a significant age difference: 50.5 years at AUSOM and 37.2 years at KNUH (*p* = 0.026). Patients without reported suicidal thoughts showed no significant age difference, with averages of 57.4 years at AUSOM and 61.1 years at KNUH (*p* = 0.281). In the AUSOM database, there were no significant differences in age, sex, medical history, and psychiatric history except for substance use disorder between the groups. The KNUH database did not differ by group overall, but the age was significantly younger in the group with suicidal thoughts.

**Table 1:** Demographic and clinical characteristics of patients with depression in AUSOM.

Characteristics	Without suicidal thoughts (*n* = 116)	With suicidal thoughts (*n* = 36)	*P*-value
Socio-demographics, *n* (%)			
Female	71 (61.2)	23 (63.9)	0.93
Race, Korean	116 (100.0)	36 (100.0)	1.00
Age, Mean (SD)	57.4 (17.7)	50.5 (17.2)	0.05
Medical history, *n* (%)			
Hypertension	21 (18.1)	9 (25.0)	0.50
Hyperlipidemia	4 (3.4)	0 (0.0)	0.59
Diabetes	4 (3.4)	1 (2.8)	1.00
Psychiatric history, *n* (%)			
Anxiety disorder	10 (8.6)	1 (2.8)	0.42
Sleep disorder	7 (6.0)	5 (13.9)	0.24
Substance use disorder	2 (1.7)	5 (13.9)	0.01*

*Indicates statistical significance (*p* < 0.05).

### Model specification

**Fig. 1:**
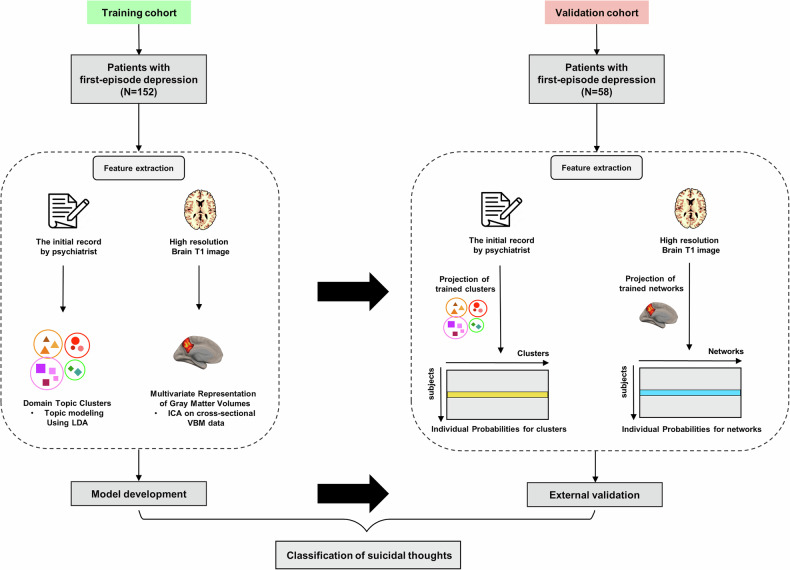
Analysis workflow of suicide classification. Selected features from NLP-derived features and brain morphometric features were used to develop suicide classification models according to combinations of data in the training cohort. After feature extractions using trained clusters and networks were performed in the validation cohort, the developed models were externally validated. NLP natural language processing, LDA Latent Dirichlet allocation, ICA independent component analysis, VBM voxel-based morphometry.

A study overview is shown in Fig. 1. The psychiatric symptoms from the psychiatrist’s initial notes at the time of depression diagnosis and brain MRI scans within 1 month of their first diagnosis were used to develop and validate the classification model.

**Fig. 2:**
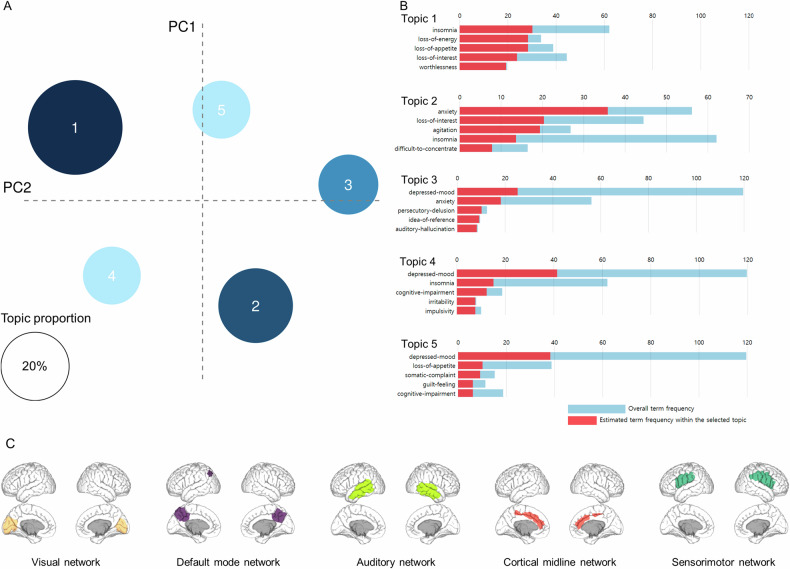
Extracted features for the classification model using NLP and source-based brain morphometry. **A** Intertopic distance map. The size of each circle indicates the topic proportions for all text. **B** Keywords representing each topic. Each red bar presents a term frequency in each topic. **C** Structural brain networks including visual network, default mode network, auditory network, cortical midline network, and sensorimotor network.

**Fig. 3:**
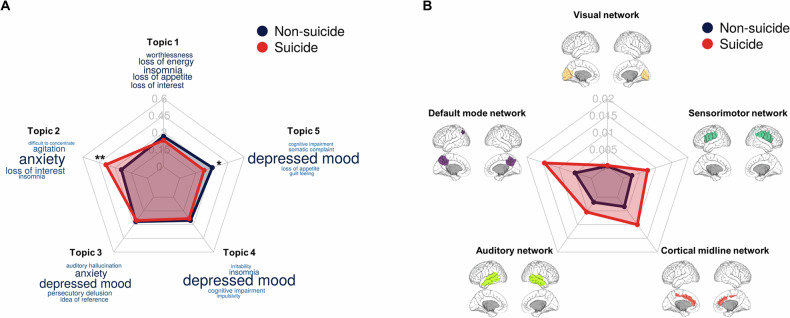
Comparison of extracted features between suicidal and non-suicidal thoughts groups. **A** Comparison of topic probability between the suicidal and non-suicidal thoughts groups. **B** Comparison of network weights between the suicidal and non-suicidal groups. *indicates statistical significance at *p* < 0.05. **Indicates statistical significance at *p* < 0.01.

Figure 2 shows the clustered topics through NLP and brain networks selected through the source-based morphometry (SBM) analysis. Through NLP, the clustered topics were neurovegetative, anxiety, psychotic, insomnia, and somatic symptoms. After SBM analysis, we selected five independent component (IC) maps that were localized at hypothetical large-scale functional brain networks related to depression [the visual network, default mode network (DMN), auditory network (AN), cortical midline network (CMN), and sensorimotor network (SMN)] [35, 36]. Figure 2 shows the specific brain regions of the five IC maps. Comparisons of the characteristics of the groups by suicide status showed that topic 2 (related to anxiety) was significantly higher and topic 5 (related to somatic symptoms) was significantly lower in the suicide group (Fig. 3). There were no significant differences in the MRI features between groups, but the proportions of the networks differed overall.

In the correlation test between MRI and text variables, all r-values were below 0.4, indicating no detectable correlation (Supplementary Fig. 4). Based on these results, all MRI and text variables were used in the model development.

### Improvement of suicide classification through the addition of brain MRI

**Fig. 4:**
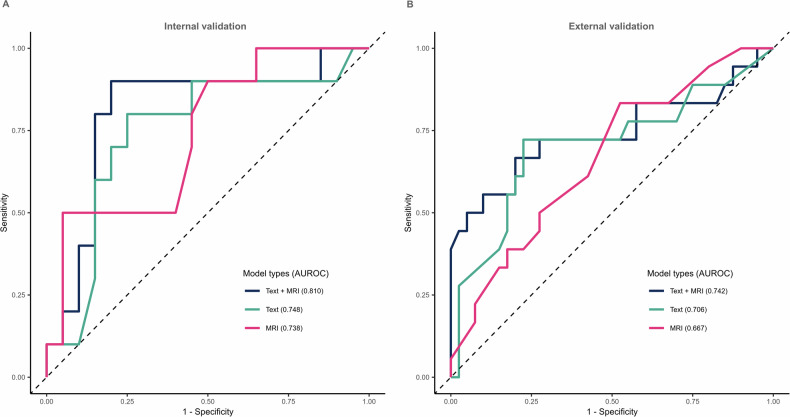
Performance of models classifying suicidal thoughts. **A** ROC curve for the models according to data combinations in the internal validation. **B** ROC curve for the models according to data combinations in the external validation. ROC Receiver operating characteristic.

The receiver operating characteristic (ROC) curves of the models obtained using XGBoost are shown in Fig. 4. The performance of the model using either text or MRI data gave area under the receiver operating characteristic curves (AUROCs) of 0.748 [95% CI: 0.544–0.951] and 0.738 [95% CI: 0.546–0.929], respectively (Table 2). In terms of AUROC, the multimodal model combining two data types showed a trend toward improvement over models with only one data type (Text + MRI: 0.810; 95% CI: 0.624–0.996). There was no significant difference in the AUROCs between the multimodal model combining two data types and the model using only MRI data (*p* = 0.304). However, there were significant differences in the AUROCs between the multimodal model combining two data types and the model using text data only (*p* = 0.037). In other evaluation metrics, the multimodal model gave the highest predictive performance (accuracy: 0.833, sensitivity: 0.900, specificity: 0.800, F1 score: 0.783) (Supplementary Table 5).

**Table 2:** AUROC comparisons using the DeLong test between prediction models.

Comparison	AUROC (95% CI)	AUROC (95% CI)	*p*-value
Internal validation			
Text vs Text + MRI	0.748 (0.544–0.951)	0.810 (0.624–0.996)	0.037*
MRI vs Text + MRI	0.738 (0.546–0.929)	0.810 (0.624–0.996)	0.304
Text vs MRI	0.748 (0.544–0.951)	0.738 (0.546–0.929)	0.474
Internal validation vs External validation			
Text + MRI (Internal vs external)	0.810 (0.624–0.996)	0.742 (0.577–0.907)	0.296
Text (Internal vs external)	0.748 (0.544–0.951)	0.706 (0.543–0.868)	0.377
MRI (Internal vs external)	0.738 (0.546–0.929)	0.667 (0.518–0.816)	0.285

*AUROC* area under the receiver operating characteristics curve, *CI* confidence interval.

*Indicates statistical significance (*p* < 0.05).

### Models using a different database performed well

The models that used the AUSOM data for development and internal validation were then externally validated using the KNUH data on a model-by-model basis (Fig. 1, Supplementary Fig. 2). The differences in the AUROCs between internal validation and external validation were not significant (Text + MRI (internal vs. external): 0.810 vs. 0.742, Text only (internal vs. external): 0.748 vs. 0.706, MRI only (internal vs. external): 0.738 vs. 0.667, respectively) (Fig. 4, Table 2). This result suggests the generalizability of the model to geographically different data. The external validation also showed that the multimodal models that combined two data types tended to outperform models with only one data type.

During the external validation, we observed a significant difference in the age of the AUSOM data and KNUH data. After excluding the effect of age from the MRI features, we checked whether the models using a different database performed as well. The method for excluding the effect of age is described in the Supplementary Method and was applied to both the AUSOM and KNUH data. After excluding the effect of age, the internal validation performance of the model using only MRI data showed an AUROC of 0.708, which was similar to the AUROC of 0.738 of the original model. The model combining the two data types improved internal validation performance with an AUROC of 0.838, similar to the original model’s AUROC of 0.810. For external validation, the combined model had an AUROC of 0.742 and the model using only MRI data had an AUROC of 0.650, similar to the original model’s AUROC of 0.742 for combined data and 0.667 for MRI only.

### Features with the greatest contributions to classification

For the multimodal model combining two data types, a graphical explanation of feature importance based on the average effect on the magnitude of the model output is shown in Supplementary Fig. 3. The graph of the Y-axis represents features in the classification model, ranked in descending order. The top predictors were topics, followed by brain MRI variables. The X-axis in the SHAP beeswarm plot shows the SHAP values. The SHAP value of each dot reflects the effect of a feature in the SHAP plot. For example, high median values of topic 2 (related to anxiety), loading weights of the DMN, loading weights of the CMN, topic 3 (related to psychosis), and topic 4 (related to insomnia) were powerful predictors of suicide. Low median values of topic 5 (related to somatic symptoms), topic 1 (related to neurovegetative symptoms), loading weights of the auditory network, loading weights of the visual network, and loading weights of the SMN were more strongly predictive of suicide.

## Discussion

In this study, we developed and externally validated an integrative model that uses MRI and text data to classify suicidal thoughts in patients with depression. Natural language processing of medical records showed that topics related to anxiety and agitation had a significantly higher frequency in the suicidal thought group, followed by topics related to depressed mood and somatic symptoms. Our SBM analysis using MRI data did not identify any significant differences in structural patterns between the suicidal thought and non-suicidal thought groups. However, there was a large difference in the mean loading weights between the two groups in the area corresponding to the DMN, especially the precuneus, followed by a difference in the area corresponding to the CMN, especially the anterior cingulate cortex (ACC). The model using clinical symptoms and structural brain patterns could classify the presence or absence of suicidal thoughts. Consistent performance across internal and external validations demonstrated our model’s generalizability. When using both data types (text and MRI), the performance was better than when using each data type individually, particularly for text data only. This is consistent with previous research findings that multimodal models outperform single-modality models [37].

Our findings showed that the AUROC was 0.810 when suicidal thoughts were classified by text and MRI combined, but the AUROC was 0.748 for text alone (*p* = 0.037). These results were consistent in the external validation and showed that the combination of MRI with clinical notes text data for evaluating suicidal thoughts in patients with depression increased the accuracy of clinical evaluation and might be broadly applicable. In other words, modeling algorithms based on both text and MRI can be used during clinical care screening to quantitatively predict the likelihood of suicidal thoughts.

A meta-analysis study that included 42 observational studies published in 2013 found that the risks of suicidal ideas, attempted suicides, completed suicides, or any suicidal behaviors were significantly higher in patients with anxiety than in patients without [38]. On the other hand, a meta-analysis study that included only longitudinal studies in 2016, found that anxiety significantly predicted suicide ideation and attempts but did not significantly predict death. The meta-analysis also found that anxiety was a weak predictor [39]. For the possible mechanism between suicide and anxiety, individuals experiencing severe anxiety, worry, and fear may consider or attempt suicide as a means to escape their distress [40]. Also, biological factors, such as reduced hydroxyindoleacetic acid levels (a metabolite of serotonin) in cerebrospinal fluid, might serve as a connection between anxiety disorders and tendencies towards suicidal behavior [41]. In the suicide model presented in a paper by Fawcett et al., dysregulation of the hypothalamic pituitary adrenal axis worsened anxiety or agitation and their interactions with depression and impulsivity increased the risk of suicide [42]. Consistent with the results of previous studies, our results confirmed that suicidal risk was high when anxiety/agitation was mainly mentioned in clinical notes.

Additionally, in cases in which somatic symptoms were the main topic along with depression, the risk of suicidal thought was higher in cases in which somatic symptoms were mentioned less. A systematic review in 2021 showed that somatic symptom disorder increased the risk of suicidal ideation and attempts independently of comorbid mental disorder [43]. Our results were contradictory possibly because of different characteristics of the participants in our study. We targeted people with depressive symptoms among psychiatric outpatients at general hospitals who visited the hospital with various symptoms such as insomnia, anxiety, and somatization symptoms. In cases in which patients receive psychiatric outpatient treatment at a general hospital but have no suicidal thoughts and undergo both MRI and clinical evaluation, somatic complaints and loss of appetite may have been the main symptoms. In other words, it is a possible that physical problems should have been ruled out. Additionally, unlike people in Western cultures, Koreans view the expression of depressive symptoms as shameful and tends to complain mainly of somatic symptoms [44]. A 2016 study of Korean patients with major depressive disorder found that somatic symptoms significantly predicted the severity of depressive symptoms [45]. From this finding, it can be inferred that the group with depressive symptoms but without suicidal thoughts visited the outpatient clinic mainly complaining of somatic symptoms.

Our SBM analysis showed that there were no significant differences in the structural network patterns between the suicidal thought and non-suicidal thought groups. The greatest difference between the groups was in the precuneus, which corresponds to the DMN network. The risk of suicide was high in the patients with a large precuneus volume. Our findings are consistent with those of previous research in which suicidal patients were found to have a larger cortical volume within the superior parietal lobe, which includes the precuneus [46]. The precuneus participates in self-centered mental imagery strategies [47], and negative self-image has been found to be associated with suicidal ideation in adolescents seeking psychiatric assessment [48]. Additionally, in a recent machine learning study using whole brain regions of interest targeting adolescents, the volumes of the superior frontal gyrus, inferior frontal gyrus, orbitofrontal cortex, superior and middle temporal gyrus, and fusiform gyrus were smaller in the group with a suicide risk than in the group without, but the volume of the precuneus was larger [49].

Other than the DMN network, although not statistically significant, the most notable difference between the suicidal thought and the non-suicidal thought groups was in the ACC region, which corresponds to the CMN. A Previous review study showed that the cortical midline structure is related to self-focused thinking and rumination, and dysfunction in this area can cause suicidal thoughts or behaviors [50]. Our results showed that as the network loading weight (a proxy for brain volume) increased, the risk of suicidal ideation also increased. Contrary to our findings, a previous review showed that suicide risk was higher in groups with smaller ACC volumes [51, 52]. However, in a study conducted on patients with type I bipolar disorder in 2017, the group with a large ACC volume had more suicide attempts and higher lethality [53]. In that article, the authors proposed that hypertrophy of ACC may occur as a compensatory mechanism for regulating emotional states [53]. In the present study, we selected participants who had chiefly complained of depression, but because the complaints were based on the first-time outpatient-visit records, bipolar disorder might not have been completely distinguished from the patients’ longitudinal course. In support of this, anxiety and agitation, which were prominent in the suicidal thought group, are among the findings suggesting bipolar disorder [54].

The strength of this study is that it not only developed a model using data from a single institution, but also validated it by applying the model to other medical institutions, proving its general applicability. The same results from two institutions with different treatment settings further ensured the reliability of the results of this study. Second, medical records and brain MRI were used to perform multimodal modeling. As an objective diagnostic method, MRI can complement the limitations of psychiatric evaluation that relies on subjective reports from patients and guardians. Moreover, unlike functional MRI, structural brain MRI does not require cumbersome tasks, and the imaging procedure is relatively simple, making it a good modality for use in a psychiatric outpatient clinic of a general hospital. Lastly, there are several benefits in terms of actual clinical utility. Because the model was developed using data assessed by trained psychiatrists in a general hospital setting, it may be well suited to identifying patients in circumstances of concealment. In addition, given that general practitioners tend to lack confidence in recognizing and managing suicidal patients [55], this predictive model could be a tool for physicians of other specialties or general practitioners to assist in the assessment of suicide. This usefulness is expected to be further doubled if our results and clinical applications are confirmed through future large-scale research.

There were several limitations in our study. First, the longitudinal outcome could not be determined because we used cross-sectional data from the first outpatient visit. However, the study purpose was to classify the presence or absence of suicidal thoughts at the first visit, and it is meaningful that appropriate results were confirmed. Second, the number of research subjects was not large enough to optimally support the learning of classification models and generalize this result. Although our sample was not particularly small compared with studies that used existing brain imaging [56], results should be interpreted with sample size limitations. Third, we used structural volumes based on T1 images rather than on various MRI sequences. However, by utilizing the image sequences most frequently captured in actual clinical settings, we were able to create a clinically useful and feasible classification model. Fourth, this study used the combined MRI data from 1.5 T and 3 T scanners. Although it is better to use the same scanner for consistency, previous studies have reported similar brain-volume patterns between 1.5 T and 3 T [57]. A way to solve the problem in this situation is harmonization, which can improve the generalizability and robustness of the model. We chose to use a non-linear algorithm instead of harmonization, but further research using harmonization is needed to validate the model. Fifth, frontotemporal and subcortical networks known to be associated with suicide were not included as key features. These networks were included in the feature extraction process but did not pass the high reliability criteria. This is probably due to insufficient patient numbers, given that frontotemporal and subcortical networks were included in studies with higher patient numbers [58]. Future studies with larger numbers of patients are needed. Sixth, we did not include psychological scales such as HAM-D and somatic symptom scores in the study. Although we had scores from psychological scales for some patients, we were unable to add criteria that included the scales due to a decreasing sample size. Lastly, this study did not distinguish between suicidal ideation and attempts in developing the model. Labeling different levels of suicidality as suicidal thoughts may result in the loss of important information. Some studies have made separate predictions for suicidal ideation and attempts [59]. However, there have been attempts to analyze suicidal ideation and attempts together, and Laura et al. showed that they are difficult to distinguish [60].

In conclusion, our study findings showed that a combination of neuroimaging and NLP variables improved the classification of suicidal thoughts over NLP measurement only. In light of our personalized approach, our study findings can be used to focus on evaluating suicidal thoughts in patients for whom anxiety or agitation is the primary complaint and the volume of the area corresponding to the DMN or the CMN is relatively large. Replication of our results by other groups using a multidimensional approach to achieve meaningful classification of suicidal thoughts is needed.

### Supplementary information

Supplementary material

## Data Availability

The data sets generated and analyzed during this study are available from the corresponding author upon reasonable request.
