# Supplementary material for: Neuroimaging and natural language processing-based classification of suicidal thoughts in major depressive disorder
Source: Transl Psychiatry. 2024 Jul 4;14:276. doi: 10.1038/s41398-024-02989-7 (PMC11224278; doi:10.1038/s41398-024-02989-7)

**Supplementary Materials**

Supplement to “Neuroimaging and Natural Language Processing-Based Classification of Suicidal Thoughts in Major Depressive Disorder”

**Supplementary Method. Regression procedure for age from MRI features**

Regression procedure (for removing the effect of age) was applied to weights of all networks (i.e., IC) with the following equation.

$$\boldsymbol{Y}_{IC weight matrix}=\boldsymbol{Xb}+\boldsymbol{E}_{error}$$

$\boldsymbol{X}=[\boldsymbol{1} \boldsymbol{x}_{\boldsymbol{age}}]$, $\boldsymbol{b}=\left[ \beta_{0} \beta_{1} \right]^{\top}$

where $\boldsymbol{Y}_{IC weight matrix}$ contains all IC weights for all subjects (*n* x *k*) and *n* and *k* each represents the number of subjects and networks included in our study. Also, $\boldsymbol{1}$ and $\boldsymbol{x}_{\boldsymbol{age}}$ are intercept (*n* x 1) and age vector (*n* x 1), $\boldsymbol{b}$ is the corresponding effects, and $\boldsymbol{E}_{error}$ is the error matrix. Then, we simply regressed out the effect of age by multiplying the well-known residual-forming matrix, $\left( \boldsymbol{I}_{\boldsymbol{n}}\boldsymbol{-}\boldsymbol{P}_{\boldsymbol{X}} \right)$, in front of $\boldsymbol{Y}_{IC weight matrix}$ as follows.

$$\boldsymbol{Y}_{IC weight matrix}^{filtered}\boldsymbol{=}\left( \boldsymbol{I}_{\boldsymbol{n}}\boldsymbol{-}\boldsymbol{P}_{\boldsymbol{X}} \right)\boldsymbol{Y}_{IC weight matrix}$$

where $\boldsymbol{I}_{n}$ is the n-dimensional identity matrix and $\boldsymbol{P}_{\boldsymbol{X}}\boldsymbol{=X}{\boldsymbol{(}\boldsymbol{X}^{\top}\boldsymbol{X)}}^{\boldsymbol{-1}}\boldsymbol{X}^{\top}$ is the projection matrix regarding $\boldsymbol{X}$.

**Supplementary Table 1. Code list for definitions**

| **Variables** | **Vocabulary** | **OMOP Vocabulary Codes** |
| --- | --- | --- |
| Depression | SNOMED | 440383 (Depressive disorder),  442306 (Adjustment disorder with depressed mood),  4175329 (Organic mood disorder of depressed type) |
| Brain MRI | SNOMED | 36713262 (MRI of brain without contrast),  36717294 (MRI of brain with contrast),  37311324 (MRI of brain) |
| Schizophrenia spectrum and bipolar disorder | SNOMED | 435783 (Schizophrenia),  4286201 (Schizoaffective disorder),  4335169 (Acute transient psychotic disorder),  35207135 (Shared psychotic disorder),  37117049 (Substance induced psychotic disorder),  434010 (Schizotypal personality disorder),  432590 (Delusional disorder),  436665 (Bipolar disorder) |
| Dementia | SNOMED | 4182210 (Dementia) |

**Supplementary Table 2. MRI scan parameters for each database**

| **Database** | **Parameters** |
| --- | --- |
| **AUSOM** |  |
| 1.5 T | TR = 500 ms, TE = 16 ms, FA (flip angle) = 72°,  FOV = 125.12 × 75.07 mm^2^, acquisition matrix = 320 × 192,  slice thickness = 5 mm |
| 3.0 T | TR = 466.67 ms, TE = 10 ms, FA (flip angle) = 75°,  FOV = 100.01 × 87.10 mm^2^, acquisition matrix = 256 × 224,  slice thickness = 6 mm |
| **KNUH** |  |
| 1.5 T | TR = 1730 ms, TE = 15 ms, FA (flip angle) = 150°,  FOV = 193 × 220 mm^2^, acquisition matrix = 320 × 280,  slice thickness = 5 mm |
| 3.0 T | TR = 2000 ms, TE = 30 ms, FA (flip angle) = 90°,  FOV = 230 × 230 mm^2^, acquisition matrix = 400 × 276,  slice thickness = 5 mm |

**Supplementary Table 3. Hyperparameters searched for the machine learning models**

| **Hyperparameter** | **Values** |
| --- | --- |
| n_estimators | 100, 200, 500, 1000 |
| max_depth | 2, 3, 4, 5 |
| colsample_bytree | 0.3, 0.4, 0.5 |
| learning_rate | 0.01, 0.02, 0.04, 0.1 |
| gamma | 0.0, 0.2, 0.4 |

**Supplementary Table 4. Demographic and clinical characteristics of patients with depression in KNUH**

| **Characteristics** | **Without suicidal thoughts (n=40)** | **With suicidal thoughts (n=18)** | **P-value** |
| --- | --- | --- | --- |
| **Socio-demographics, n (%)** |  |  |  |
| Female | 25 (62.5) | 7 (38.9) | 0.17 |
| Race, Korean | 40 (100.0) | 18 (100.0) | 1.00 |
| **Age, Mean (SD)** | 61.1 (19.9) | 37.2 (22.4) | <0.01* |
| **Medical history, n (%)** |  |  |  |
| Hypertension | 4 (10.0) | 2 (11.1) | 1.00 |
| Hyperlipidemia | 9 (22.5) | 1 (5.5) | 0.23 |
| Diabetes | 1 (2.5) | 1 (0.0) | 1.00 |
| **Psychiatric history, n (%)** |  |  |  |
| Anxiety disorder | 7 (17.5) | 5 (27.8) | 0.59 |
| Sleep disorder | 2 (5.0) | 1 (5.5) | 1.00 |
| Substance use disorder | 2 (5.0) | 0 (0.0) | 0.85 |

* indicates statistical significance (p < 0.05).

**Supplementary Table 5. Performance results of prediction models**

| **Performance metrics** | **Internal validation** | | | **External validation** | | |
| --- | --- | --- | --- | --- | --- | --- |
|  | **Text + MRI** | **Text** | **MRI** | **Text + MRI** | **Text** | **MRI** |
| ACC | 0.833 | 0.767 | 0.800 | 0.759 | 0.758 | 0.586 |
| Sensitivity | 0.900 | 0.800 | 0.500 | 0.667 | 0.722 | 0.833 |
| Specificity | 0.800 | 0.750 | 0.950 | 0.800 | 0.775 | 0.475 |
| AUROC | 0.810 | 0.748 | 0.738 | 0.742 | 0.706 | 0.667 |
| F1 score | 0.783 | 0.696 | 0.625 | 0.632 | 0.650 | 0.556 |

*Notes*: ACC, accuracy; AUROC, area under the receiver operating characteristics curve

**Supplementary Table 6. Performance results of prediction models after excluding the effect of age**

| **Performance metrics** | **Internal validation** | | **External validation** | |
| --- | --- | --- | --- | --- |
|  | **Text + MRI** | **MRI** | **Text + MRI** | **MRI** |
| ACC | 0.800 | 0.767 | 0.793 | 0.603 |
| Sensitivity | 0.900 | 0.400 | 0.611 | 0.667 |
| Specificity | 0.750 | 0.950 | 0.875 | 0.575 |
| AUROC | 0.838 | 0.708 | 0.742 | 0.650 |
| F1 score | 0.750 | 0.533 | 0.647 | 0.511 |

*Notes*: ACC, accuracy; AUROC, area under the receiver operating characteristics curve

Supplementary Figure 1. The perplexity graphs which indicate the optimal number of LDA topics


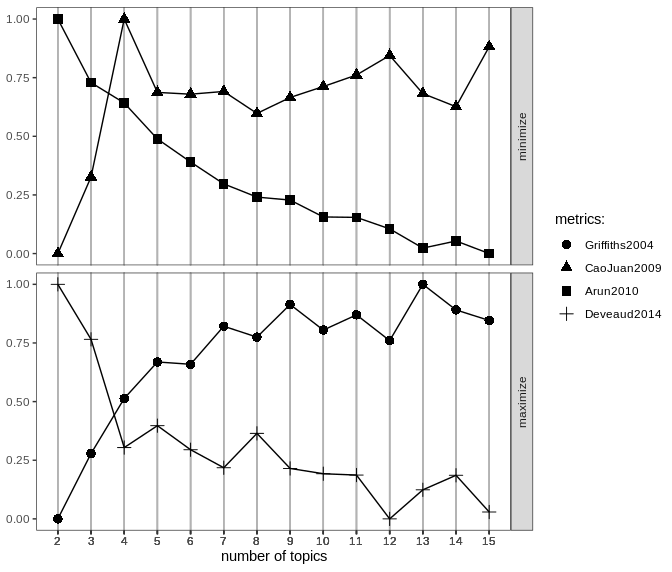


Supplementary Figure 2. Feature extraction leveraging brain morphometry and natural language processing in training and validation cohorts


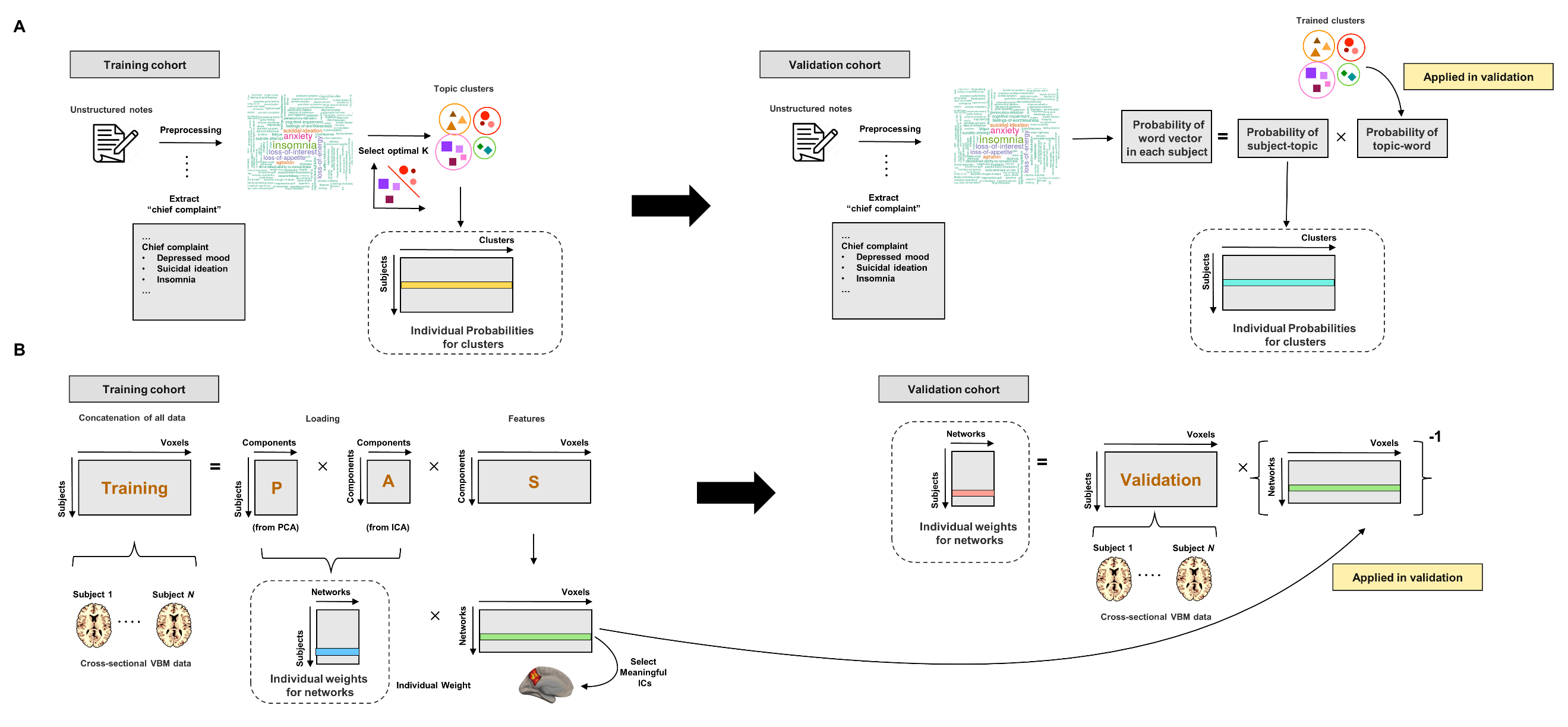


Supplementary Figure 3. SHAP beeswarm plot of the prediction model using all types of data


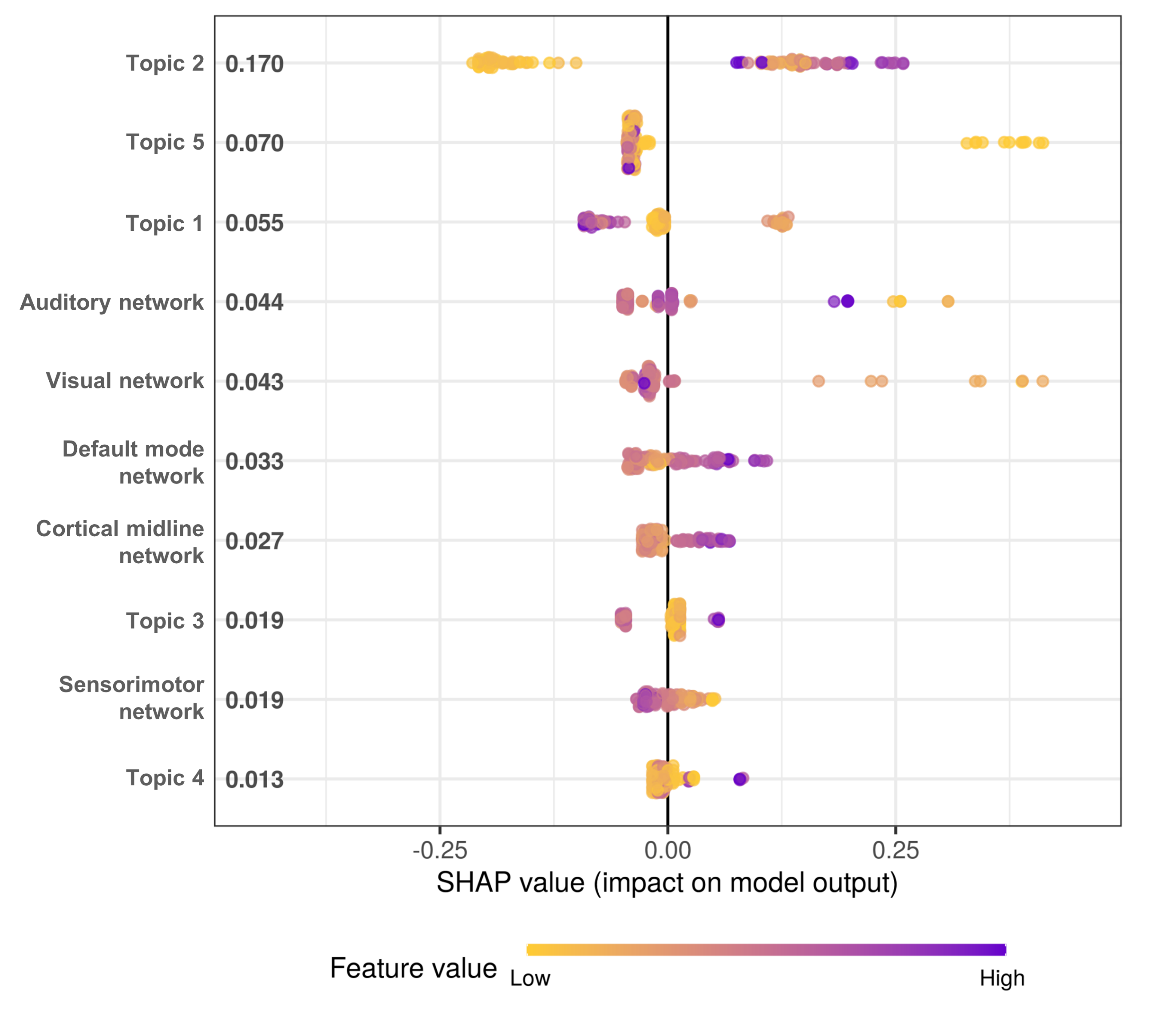


Supplementary Figure 4. Pearson product-moment correlations between MRI variables and text variables


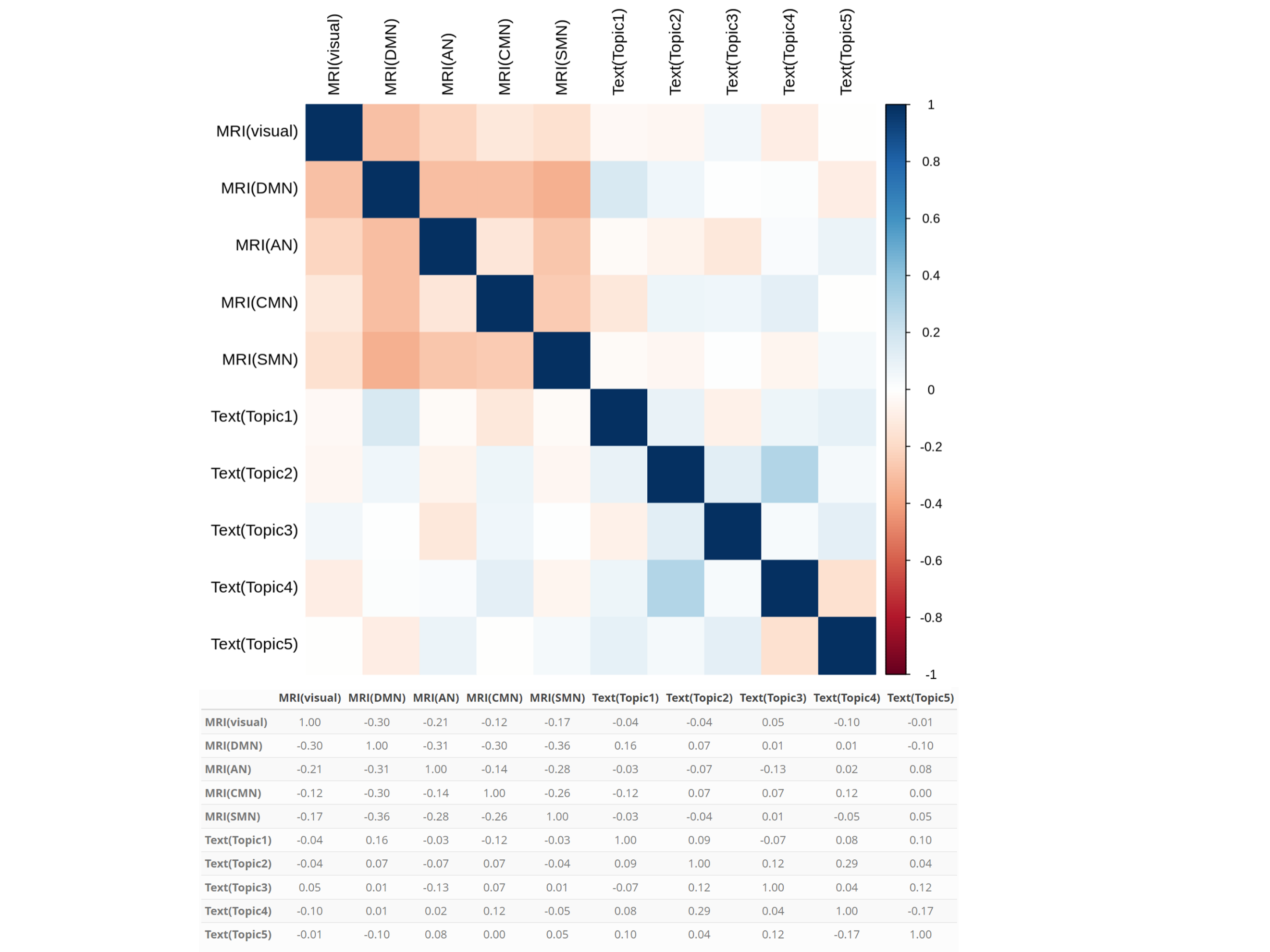

Supplement: Supplementary file 1 — Supplementary material [file 41398_2024_2989_MOESM1_ESM.docx]
